# An Enzymatic Atavist Revealed in Dual Pathways for Water Activation 

**DOI:** 10.1371/journal.pbio.0060206

**Published:** 2008-08-26

**Authors:** Donghong Min, Helen R Josephine, Hongzhi Li, Clemens Lakner, Iain S MacPherson, Gavin J. P Naylor, David Swofford, Lizbeth Hedstrom, Wei Yang

**Affiliations:** 1 School of Computational Science, Florida State University, Tallahassee, Florida, United States of America; 2 Department of Biochemistry, Brandeis University, Waltham, Massachusetts, United States of America; 3 Department of Biological Sciences, Florida State University, Tallahassee, Florida, United States of America; 4 Department of Chemistry, Brandeis University, Waltham, Massachusetts, United States of America; 5 Department of Chemistry and Biochemistry, Florida State University, Tallahassee, Florida, United States of America; 6 Institute of Molecular Biophysics, Florida State University, Tallahassee, Florida, United States of America; 7 College of Life Science, Nankai University, Tianjin 300071, China; Stanford University, United States of America

## Abstract

Inosine monophosphate dehydrogenase (IMPDH) catalyzes an essential step in the biosynthesis of guanine nucleotides. This reaction involves two different chemical transformations, an NAD-linked redox reaction and a hydrolase reaction, that utilize mutually exclusive protein conformations with distinct catalytic residues. How did Nature construct such a complicated catalyst? Here we employ a “Wang-Landau” metadynamics algorithm in hybrid quantum mechanical/molecular mechanical (QM/MM) simulations to investigate the mechanism of the hydrolase reaction. These simulations show that the lowest energy pathway utilizes Arg418 as the base that activates water, in remarkable agreement with previous experiments. Surprisingly, the simulations also reveal a second pathway for water activation involving a proton relay from Thr321 to Glu431. The energy barrier for the Thr321 pathway is similar to the barrier observed experimentally when Arg418 is removed by mutation. The Thr321 pathway dominates at low pH when Arg418 is protonated, which predicts that the substitution of Glu431 with Gln will shift the pH-rate profile to the right. This prediction is confirmed in subsequent experiments. Phylogenetic analysis suggests that the Thr321 pathway was present in the ancestral enzyme, but was lost when the eukaryotic lineage diverged. We propose that the primordial IMPDH utilized the Thr321 pathway exclusively, and that this mechanism became obsolete when the more sophisticated catalytic machinery of the Arg418 pathway was installed. Thus, our simulations provide an unanticipated window into the evolution of a complex enzyme.

## Author Summary

Many enzymes have the remarkable ability to catalyze several different chemical transformations. For example, IMP dehydrogenase catalyzes both an NAD-linked redox reaction and a hydrolase reaction. These reactions utilize distinct catalytic residues and protein conformations. How did Nature construct such a complicated catalyst? While using computational methods to investigate the mechanism of the hydrolase reaction, we have discovered that IMP dehydrogenase contains two sets of catalytic residues to activate water. Importantly, the simulations are in good agreement with previous experimental observations and are further validated by subsequent experiments. Phylogenetic analysis suggests that the simpler, less efficient catalytic machinery was present in the ancestral enzyme, but was lost when the eukaryotic lineage diverged. We propose that the primordial IMP dehydrogenase utilized the less efficient machinery exclusively, and that this mechanism became obsolete when the more sophisticated catalytic machinery evolved. The presence of the less efficient machinery could facilitate adaptation, making the evolutionary challenge of the IMPDH reaction much less formidable. Thus our simulations provide an unanticipated window into the evolution of a complex enzyme.

## Abstract 3

How does nature construct complex catalysts? Molecular simulations revealed two sets of catalytic residues in the enzyme IMPDH, one of which seems to represent a primitive catalytic machinery that may be a vestige of evolution.

## Introduction

Textbooks extol the extraordinary catalytic power and specificity of enzymes, yet the ability of many enzymes to promote several different chemical transformations is even more remarkable. In examples such as the polyketide synthases, the substrate is tethered to a flexible linker and swings gymnastically between separate active sites [1]. The evolutionary path to the assembly of such enzymes seems reasonably straightforward: gene duplication and recombination, followed by optimization of a promiscuous activity [2–6]. In contrast, enzymes such as IMP dehydrogenase (IMPDH) move around a stationary substrate, restructuring the active site to accommodate different transition states [7]. Such enzymes pose an evolutionary conundrum: it seems unlikely that Nature could simultaneously install multiple sets of catalytic machinery into the ancestral protein. IMPDH controls the entry of purines into the guanine nucleotide pool, which suggests that the origins of IMPDH are primordial, so the ancestral IMPDH probably utilized a simpler catalytic strategy.

**Figure 1:**
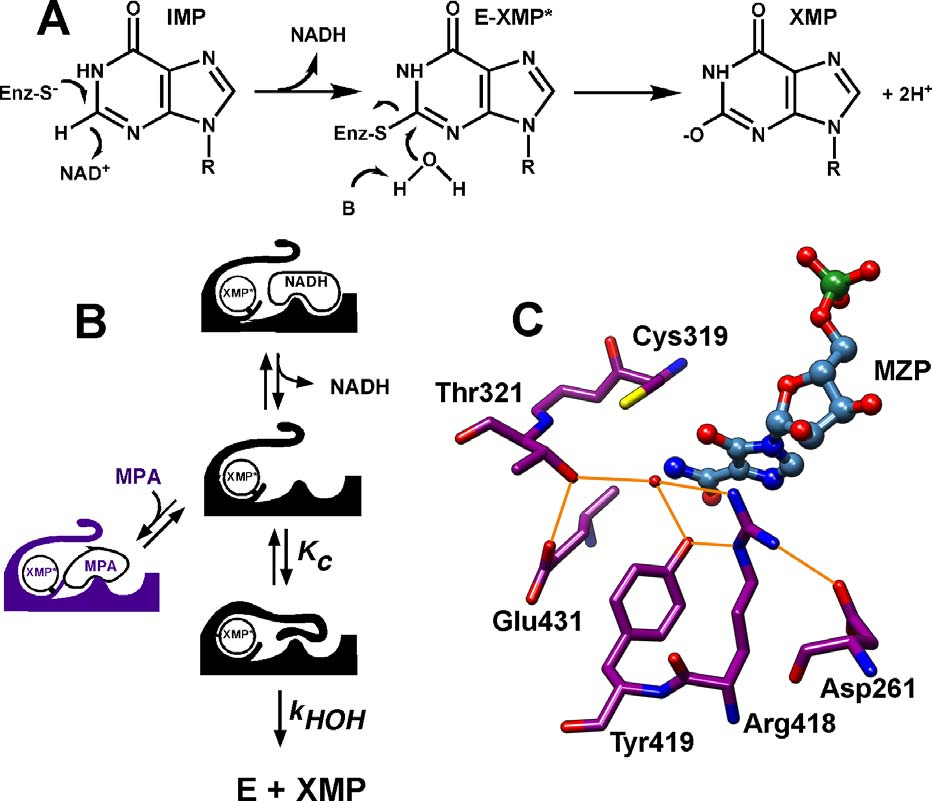
The Mechanism of IMPDH (A) The reaction involves the initial conversion of IMP to E-XMP* with concomitant reduction of NAD^+^, followed by the hydrolysis of E-XMP* to produce XMP. (B) The conformational changes in the IMPDH catalytic cycle: a protein flap moves into the dinucleotide site after NADH departs, forming the closed conformation required for the hydrolysis reaction. (C) The E•mizoribine monophosphate (MZP) complex identifies a likely hydrolytic water that interacts with Thr321, Arg418, and Tyr419 (PDB accession number 1PVN [9]).

IMPDH catalyzes two very different chemical transformations: (1) a dehydrogenase reaction between IMP and NAD^+^ that produces a Cys319-linked intermediate E-XMP* and NADH, and (2) a hydrolysis reaction that releases XMP (Figure 1A) [7,8]. A mobile flap is open during the hydride transfer reaction, permitting the association of NAD^+^. After NADH departs, this flap occupies the dinucleotide site, carrying Arg418 and Tyr419 into the active site and converting the enzyme into a hydrolase (Figure 1B). Thus, the dehydrogenase and hydrolase reactions utilize mutually exclusive conformations of the active site.

All enzymes that catalyze hydrolysis reactions have some strategy to activate water. This strategy has been difficult to recognize in IMPDH because the hydrolytic water interacts with three residues that are usually protonated at physiological pH: Thr321, Arg418, and Tyr419 (Figure 1C) [9]. The rate of the hydrolysis step decreases by a factor of 10^3^ when Arg418 is substituted with Ala or Gln, whereas a decrease of approximately 20 is observed when Tyr419 is substituted with Phe [10,11]. Neither Arg418 nor Tyr419 is involved in the dehydrogenase reaction, as expected, given their position on the mobile flap. In contrast, Thr321 is found on the same loop as the catalytic Cys319, and both the dehydrogenase and hydrolysis reactions are decreased by a factor of 20 when this residue is substituted [11]. These observations suggest that Arg418 is the most likely candidate for the role of general base in the IMPDH reaction [11,12].

We performed a series of hybrid quantum mechanical/molecular mechanical (QM/MM) simulations to further investigate the mechanism of the hydrolysis reaction of IMPDH. Surprisingly, these simulations find that IMPDH possesses two mechanisms to activate water: the Arg418 pathway as previously proposed, and a second pathway utilizing Thr321. Phylogenetic analysis indicates that the Thr321 pathway was present in the ancestral enzyme. These observations suggest that the primordial IMPDH used the Thr321 pathway exclusively, and elimination of the Arg418 pathway by mutation of modern IMPDH creates an enzymatic atavist.

## Results and Discussion

**Figure 2:**
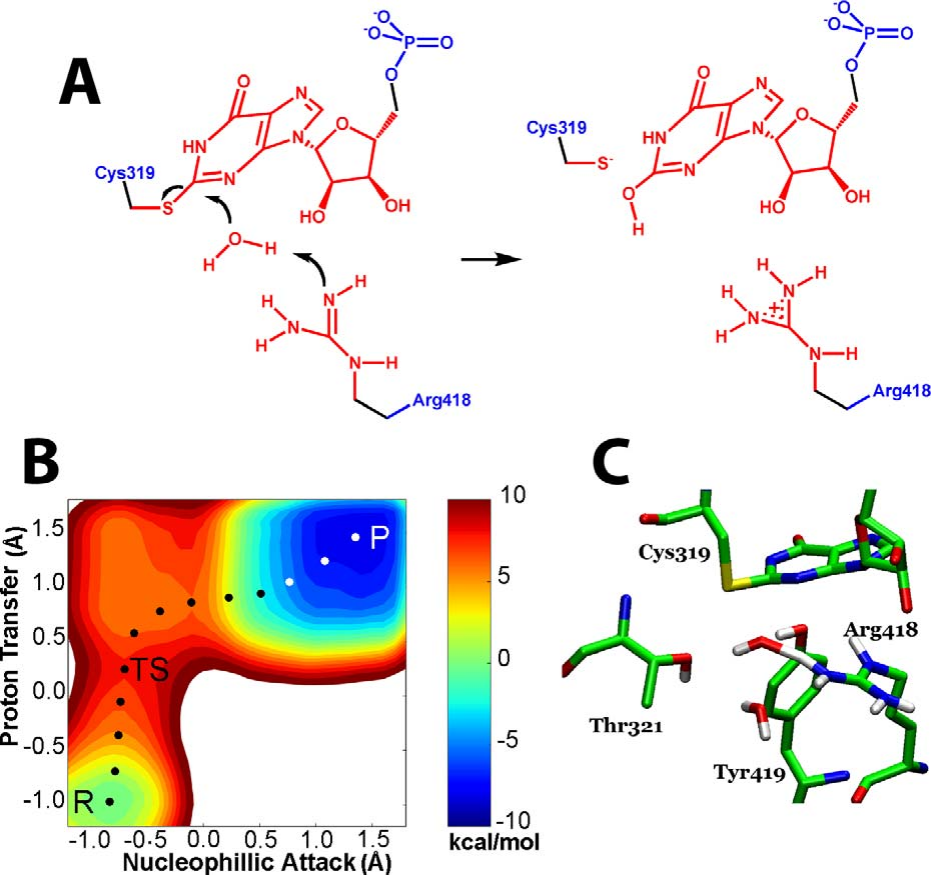
Simulation of the Arg Pathway (A) The hydrolysis of E-XMP* with Arg418 acting as the general base catalyst. Red denotes atoms treated with QM; blue denotes atoms treated with MM. (B) The free energy landscape for the Arg418 pathway. The *x*-axis denotes the difference between the distances of the migrating proton between the hydrolytic water and the NH group of Arg418, where 0.0 is the midpoint between the two acceptors; the *y*-axis specifies the progress of nucleophillic attack, where 0.0 is the midpoint between the original position of the nucleophillic oxygen and the final position. The transition state is the highest point in the energy landscape. Here, the proton has moved past the midpoint and is now associated with Arg418. In contrast, nucleophillic attack has yet to begin. P, product; R, reactant; TS, transition state. (C) The transition state structure for the Arg418 pathway.

**Figure 3:**
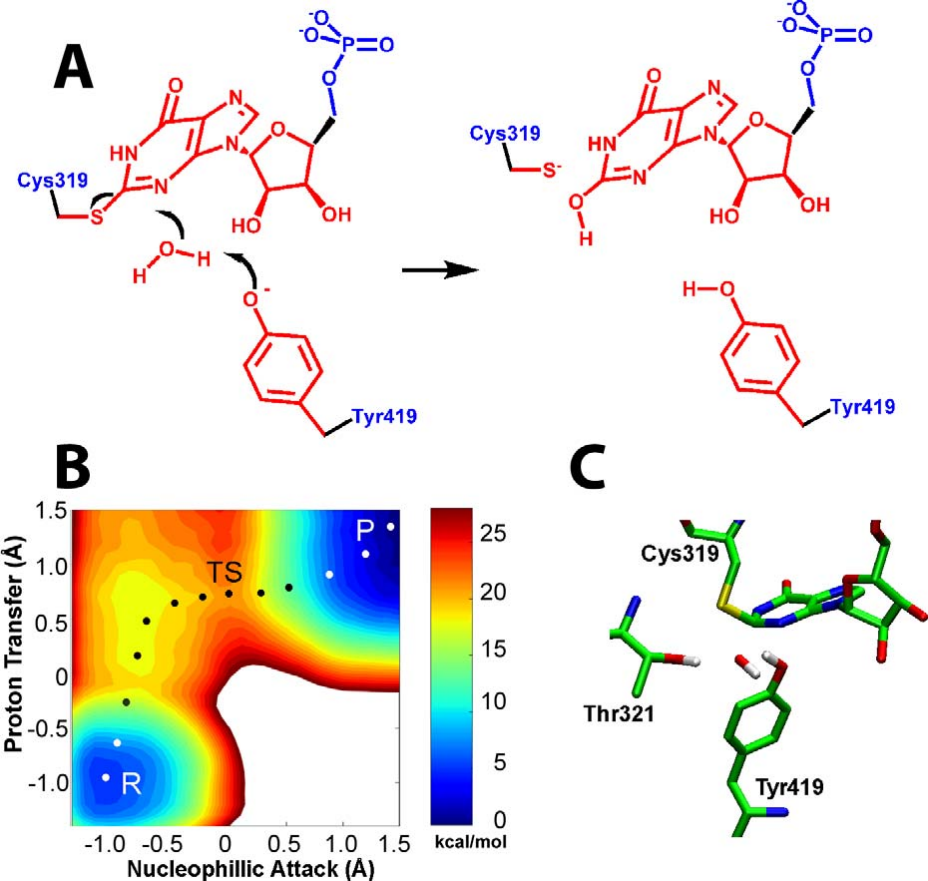
Simulation of the Tyr419 Pathway in Arg418Gln (A) The hydrolysis of E-XMP* with Tyr419 acting as the general base catalyst. Color key as described in Figure 1. (B) The free energy landscape of the Tyr419 pathway in the Ar418Gln variant, with axes as described Figure 1. P, product; R, reactant; TS, transition state. (C) The corresponding transition state structure.

**Figure 4:**
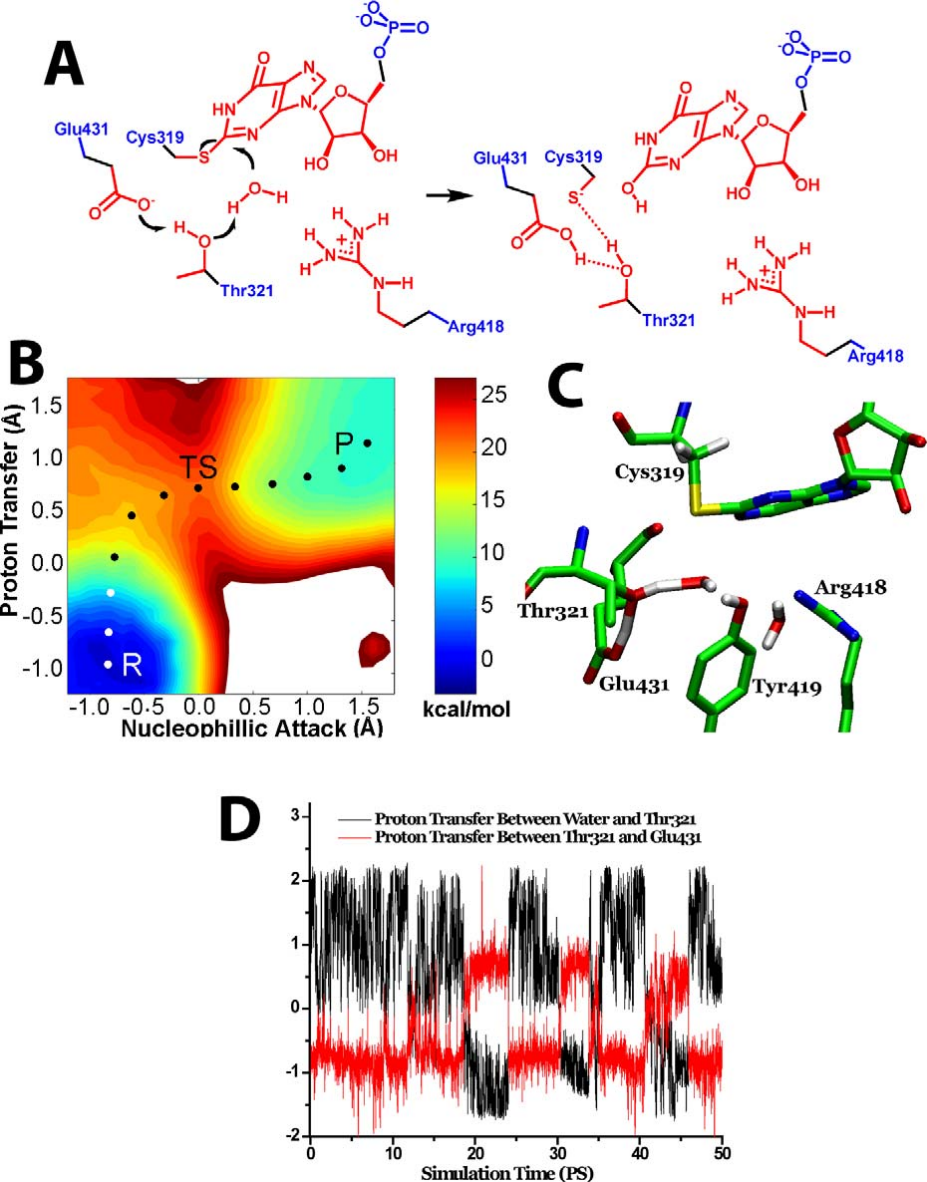
Simulation of the Thr321 Pathway (A) The hydrolysis of E-XMP* with Thr321 acting as the general base catalyst. Color key as described above. (B) The free energy landscape of the Thr321 pathway, with axes as described above, except that the second proton acceptor is the OH of Thr321. As above, proton transfer is virtually complete at the transition state, whereas nucleophillic attack has just reached the reaction midpoint. P, product; R, reactant; TS, transition state. (C) The corresponding transition state structure. (D) The correlation between proton transfer from water to Thr321 and proton transfer from Thr321 and Glu431. Atoms treated as described in Figure 1. The reaction coordinate for the proton transfer between water and Thr321 was set as the distance traversed by the proton as it moves between the oxygen of water to the oxygen of Thr321; the reaction coordinate for the proton transfer between Thr321 and Glu431 was set as the distance traversed by the proton that moves between the oxygen of Thr321 and the oxygen of Glu431.

We have applied computational methods to further investigate the mechanism of water activation in IMPDH, employing a “Wang-Landau” metadynamics algorithm [13] in hybrid quantum mechanical/molecular mechanical (QM/MM) simulations [14–21]. The Wang-Landau recursion procedure adaptively updates the height of the basis Gaussian, which allows the metadynamics algorithm to be realized in a more robust and efficient fashion. The simulation models were derived from the crystal structure of Tritrichomonas foetus IMPDH in complex with mizoribine monophosphate (MZP) (Protein Data Bank [PDB] accession code 1PVN), which describes the closed conformation of the hydrolysis reaction [9]. E-XMP* was modeled based on the guanine residue topology and parameters in CHARMM 22 [22]. The atoms in the reaction centers (colored in red in Figures 2–4) were treated quantum mechanically using the self-consistent charge density-functional tight-binding (SCCDFTB) potential [23], and the rest of the system (colored in blue in Figures 2–4) was treated classically using the CHARMM 22 force field [22]. The molecular dynamics simulations were performed with generalized solvent boundary conditions (GSBP) [24,25]. Further details on the simulations are provided in Figure S1.

### Simulation of the Hydrolysis Reaction When Arg418 Is Neutral

When Arg418 is deprotonated in the starting condition, the lowest energy pathway for the hydrolysis reaction involves the transfer of a proton to the neutral Arg418 (the Arg418 pathway, Figure 2A–2C). Proton transfer is virtually complete at the transition state, and the developing hydroxide is stabilized by interactions with Tyr419, Thr321, and another water molecule. Importantly, a stable hydroxide intermediate is not observed; the developing hydroxide instantaneously reacts with E-XMP*. These results are in remarkable agreement with experimental observations: solvent isotope effects (SIE) demonstrate that proton transfer is rate limiting (SIE = ∼2 [11]), and Bronsted analysis indicates that proton transfer is virtually complete in the transition state (β = ∼1 [12]). However, the calculated energy barrier is only 8.0 kcal/mol, much less than the experimentally observed barrier of 16 kcal/mol [10]. This difference may reflect uncertainties in the calculation, but we believe this is unlikely. A more intriguing source of discrepancy arises from the starting condition of neutral Arg418; if only a small fraction of the enzyme exists in this state, the energy barrier will be correspondingly increased. Indeed, if the pK_a_ of Arg418 is 12.5, as for a typical Arg residue, the barrier would be increased by approximately 6 kcal/mol.

### Tyr419 May Be a Surrogate General Base in the Absence of Arg418

The pK_a_ of a Tyr residue is usually two units lower than an Arg, which suggests that a deprotonated Tyr419 might activate water while Arg418 remains protonated. Further simulations argue against such a mechanism; instead, the deprotonated, negatively charged Tyr419 interacts strongly with positively charged Arg418 and cannot interact with water. Therefore, Tyr419 is unlikely to play the role of general base in the wild-type enzyme. However, the situation changes when Arg418 is substituted with Gln: now the Tyr419 phenolate can accept a proton from water. The barrier is approximately 17 kcal/mol (Figure 3). Assuming a pK_a_ of 10, as is usual for a Tyr residue, then deprotonation of Tyr419 will further increase the barrier to 21–22 kcal/mol, which is very similar to the barrier observed in the reactions of the Arg418Gln and Arg418Ala variants (∼20 kcal/mol [10,11]). As above, the simulations suggest that proton transfer is rate limiting and essentially complete in the transition state. Whereas the landscape contains a shallow valley suggesting the presence of a hydroxide intermediate, the barriers are less than a kT, so the intermediate would not have a finite lifetime. This simulation is generally consistent with experiments, where SIEs of 3–5 are observed when Arg418 is substituted [11]. However, the magnitude of these SIEs is greater than expected if the transition state is indeed late as suggested by the simulations. Interestingly, no activity is observed in the Arg418Ala/Tyr419Phe double mutant, though this fact may equally well be attributed to the inability to form the closed conformation required for the hydrolysis reaction as to the loss of the general base catalyst [12]. Together, the simulations and experiments suggest Tyr419 may act as a surrogate general base in the absence of Arg418.

Similar surrogate residues have been invoked to explain residual activity in other enzyme systems [26]. In RNase T1, His40 residue assumes the role the general base when Glu58 is substituted with Ala [27]. Similarly, in ketosteroid isomerase, Asp99 may catalyze proton transfers in the Asp38Ala variant [28]. Water or buffer molecules can also replace the function of missing catalytic residues [29,30]. These examples illustrate the resilience and plasticity of enzyme catalysis.

### Simulation of the Hydrolysis Reaction When Arg418 Is Protonated

Surprisingly, the simulations suggest a second pathway for water activation when the starting condition is protonated Arg418: Thr321 abstracts a proton from water while simultaneously transferring its own proton to Glu431 (Figure 4). As in the Arg418 pathway, the developing hydroxide is stabilized by Tyr419 and another water molecule, and the hydroxide attack occurs instantaneously; the protonated Arg418 also stabilizes the developing hydroxide by 1–2 kcal/mol. The calculated free energy barrier for the Thr321 pathway is approximately 20 kcal/mol. The proton transfers are simultaneous and rate limiting. When a simulation was performed with Glu431 treated as a molecular mechanical (MM) residue, which eliminates the possibility of proton transfer while maintaining electrostatic interactions, the energy barrier increases to at least 35 kcal/mol (Figure S2). Likewise, when Glu431 is substituted with Gln, the barrier increases to at least 38 kcal/mol. Therefore, the presence of Glu431 is essential for the operation of the Thr321 pathway.

### Experimental Verification of the Thr321 Pathway

**Figure 5:**
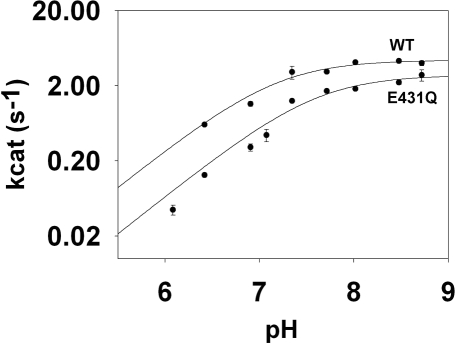
The pH Dependence of *k*_cat_ for Wild-Type and Glu431Gln IMPDH This experiment monitored the reaction with APAD^+^ instead of NAD^+^ because the hydrolysis reaction is completely rate-limiting for this dinucleotide, simplifying analysis [31]. The error bars indicate the standard errors on the values of *k*_cat_.

The simulations suggest that the Thr321 pathway is favored at low pH, whereas the Arg418 pathway becomes dominant at high pH, which predicts that the pH-rate profile will shift to the right when the Thr321 pathway is disrupted by the Glu431Gln mutation. This prediction was confirmed experimentally (Figure 5): the Glu431Gln mutation shifts the pK_a_ from 7.2 ± 0.1 to 7.6 ± 0.1, but has only a small effect on the pH-independent value of *k*_cat_ (*k*_cat_ = 2.2 and 1.4 s^−1^ for wild type and Glu431Gln, respectively; these values are in good agreement with previous reports [31,32]). Assuming that the pK_a_ shift is entirely attributable to the loss of the Thr321 pathway, the barrier for the Thr321 pathway is approximately 19 kcal/mol, as predicted by the simulations (see Figure S3).

When Arg418 is substituted with Gln, the barrier for the Thr321 pathway is approximately 21 kcal/mol, which is similar to the barrier observed experimentally in the Arg418Ala and Arg418Gln variants [10,11]. Therefore, both the Thr321 pathway and the Tyr419 pathway can account for the residual activity of the Arg418Ala and Arg418Gln variants. However, since the Thr321 pathway involves the simultaneous transfer of two protons, this pathway can account for the large solvent isotope effects observed in the Arg418 variants (SIE = 3–5 [10,11]). Therefore, we constructed the Arg418Gln/Glu431Gln variant, which should disrupt the Thr321 pathway but leave the Tyr419 pathway intact. The simulations predict that the activity of this variant should be approximately the same as the Arg418Gln, but that the solvent isotope effect should be reduced. These predictions were confirmed in subsequent experiments: (1) the value of *k*_cat_ for Arg418Gln/Glu431Gln is decreased by 50% relative to that of Arg418Gln, as expected if the Thr321 pathway was lost (0.0020 ± 0.0002 s^−1^ and 0.0040 ± 0.0004 s^−1^, respectively); and (2) though the errors on the SIE are larger than ideal, nonetheless, a smaller SIE is observed in the reaction of Arg418Gln/Glu431Gln, consistent with the loss of the Thr321 pathway (SIE = 2.1 ± 0.3 and 2.3 ± 0.4 for Arg418Gln/Glu431Gln in two independent determinations versus 2.9 ± 0.5 for Arg418Gln and 5 ± 2 for Arg418Ala [10,11]). These experiments confirm the operation of the Thr321 pathway in IMPDH.

### GMP Reductase and the Evolutionary Origins of the Thr321 and Arg418 Pathways

**Figure 6:**
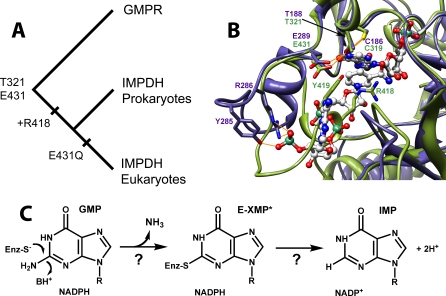
The Evolution of the IMPDH/GMPR Family (A) Evolutionary tree for IMPDH and GMPR (midpoint rooting). Substitution Glu431Gln has also occurred several times independently in bacterial IMPDH lineages (see Figure S4 and Text S1). (B) Active sites of IMPDH and GMPR. The closed conformation as observed in the X-ray crystal structure of the E•MZP complex of T. foetus IMPDH (PDB accession number 1pvn [9]) is shown in olive green, with MZP in charcoal. The E•IMP•NADPH complex of human GMPR2 (PDB accession code 2c6q) is shown in slate blue, with IMP and NADPH in gray. N, O, and P atoms are colored blue, red, and sea green, respectively. Potential hydrogen bonds are depicted in gold. The following residues were omitted for clarity: IMPDH, 57–68, 324–328, and 389–393; GMPR, 57–63, 191–195, and 250–256. This figure was rendered with Chimera [53]. (C) The GMPR reaction.

To the best of our knowledge, the presence of dual mechanisms for water activation in an enzyme active site is unprecedented. Why would an enzyme have two pathways to accomplish the same task? We believe the Thr321 pathway may be vestige of evolution, and phylogenetic analysis is consistent with this hypothesis (Figure 6; see Figure S4 for the complete phylogenetic tree). The closest relative of IMPDH is GMP reductase (GMPR), which catalyzes the conversion of GMP to IMP and ammonia with concomitant oxidation of NADPH (Figure 6) [33]. Cys319, Thr321, and Glu341 are also conserved in GMPR, which suggests that these residues were present in the IMPDH/GMPR ancestor. X-ray crystal structures show that the conserved Cys, Thr, and Glu display similar interactions in both GMPR and IMPDH (Figure 6), suggesting that these residues may have similar functions in both enzymes.

**Figure 7:**
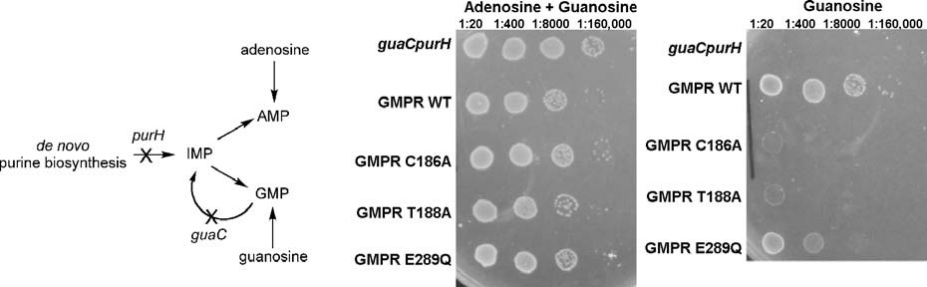
The Conserved Cys, Thr, and Glu Are Important for GMPR Activity The scheme depicts the effects of the *purH* and *guaC* mutations on the biosynthesis of adenine and guanine nucleotides. E. coli strain H1173 (*purH guaC*) requires both adenosine and guanosine for growth. The bacteria can grow on guanosine alone when transformed with a plasmid containing the wild-type (WT) *guaC* gene expressing GMPR. However, this growth is compromised when the bacteria are transformed with plasmids expressing the Cys186Ala, Thr189Ala, and Glu289Gln.

To confirm that GMPR activity depends on the presence of Cys186, Thr188, and Glu289, we tested the effect of mutations of these residues on the activity of Escherichia coli GMPR in a complementation assay (Figure 7). E. coli H1173 requires both adenosine and guanosine for growth due to mutations in both *purA* and *guaC* [34] (Figure 7). Growth on guanosine alone is restored with plasmid pGS682, which carries the wild-type *E. coli guaC* gene [35]. However, mutations in Cys186, Thr188, and Glu289 clearly compromise the ability of pGS682 to restore GMPR activity, demonstrating that selective pressure exists to conserve these residues.

Although the mechanism of the GMPR reaction has not been characterized, some clear parallels can be drawn with the IMPDH reaction, and E-XMP* may well be an intermediate. Importantly, if E-XMP* forms as proposed, the active site must be constructed to prevent the hydrolysis reaction. Kinetic and structural experiments clearly indicate that the reaction only proceeds when NADPH is bound in the active site and can block the access of water [33,36,37]. Moreover, GMPR does not contain the Arg418-Tyr419 dyad, and the flap is truncated relative to the corresponding region of IMPDH, as expected, given that the hydrolysis of E-XMP* must be avoided during the GMPR reaction. Therefore, the Arg418-Tyr419 dyad could have been installed as IMPDH optimized. Alternatively, the dyad may have been present in the ancestral IMPDH/GMPR, but was subsequently remodeled in the GMPR lineage; since the flap binds in the same site as NAD^+^, this scenario suggests that the ancestral IMPDH/GMPR was a hydrolase. While we cannot rule out the latter scenario, we note that IMPDH is a member of the FMN oxidoreductase superfamily of (β/α)_8_ barrel proteins (unfortunately, none of these proteins is sufficiently similar to permit rooting of the tree) [38–40]. Therefore, it seems more likely that the ancestral enzyme was a promiscuous dehydrogenase, and the flap carrying the hydrolase activity was the later addition.

In contrast, the Thr321 pathway was likely present in the ancestral IMPDH/GMPR. All IMPDHs and GMPRs contain Thr321 (Figures 6 and S4, and Text S1). As noted above, Thr321 also plays a role in the dehydrogenase reaction of IMPDH [11], which suggests that Thr321, like Cys319, was inherited from the ancestral redox enzyme. Glu431 is conserved among GMPRs, suggesting that the Thr321 pathway has a crucial function in this reaction, perhaps operating in the reverse to protonate the ammonia leaving group. Curiously, although Glu431 is highly conserved among IMPDHs, it is substituted with Gln in the eukaryotic branch as well as in a few prokaryotic IMPDHs. We suggest that the ancestral IMPDH/GMPR utilized the Thr321 pathway exclusively, but this pathway became expendable once the Arg418 pathway was established. Phylogenetic analysis is consistent with this view: maximum likelihood analysis indicates that the ancestral enzyme almost certainly contained Glu at position 431 (probability = 0.87) [41].

Why then is Glu431 conserved in the majority of prokaryotic IMPDHs? The presence of the Thr pathway increases turnover, which may be important in maintaining the high concentration of guanine nucleotides required to support the rapid proliferation of prokaryotes. More intriguingly, Glu431 provides 5–10-fold resistance to mycophenolic acid, a natural product that specifically inhibits IMPDH [32]. Approximately 5% of microorganisms contain some mechanism to modify mycophenolic acid, which suggests that this compound is reasonably prevalent in the environment [42]. Indeed, the extraordinary divergence of the adenosine subsite of IMPDH may be a response to the assault of natural product inhibitors such as mycophenolic acid and mizoribine [43]. This divergence occurs despite the multiple functional constraints imposed by interactions with both NAD^+^/NADH and the flap. The presence of the Thr pathway could facilitate this adaptation, making the evolutionary challenge of the IMPDH reaction much less formidable.

## Materials and Methods

### Materials.

Plasmid pGS682, a pUC plasmid carrying the 1.4-kb *guaC* insert from pGS89 [35], was a generous gift from Simon Andrews (University of Sheffield). E. coli strain H1173 was obtained from the E. coli Genetic Stock Center (Yale University).

### Computational methods.

Atoms within a radius of 22 Å around the reaction center were treated as the dynamic region; this region was propagated with regular Newtonian dynamics by applying leapfrog integrator and 1-fs time step. The atoms in the layer between the radii of 22 Å and 25 Å were treated as the buffer region; the heavy atoms in this region were harmonically restrained with the force constants scaled linearly with the distance from the sphere center. The force constants around the boundary of the 25 Å sphere were set as implied by the B factors of the crystal structure. In the buffer region, Langevin dynamics were applied with the friction coefficients also linearly scaled with the distance from the sphere center. The friction coefficients around the boundary 25 Å sphere were set as 60. CHARMM 22 force fields [22] were utilized as the molecular mechanical potentials in these simulations (colored in blue in Figures 2–4) and SCCDFTB (self-consistent charge density-functional tight-binding) method was applied as the quantum mechanical potential on the atoms involved in the chemical reactions (colored in red in Figures 2–4). For the nonbonded interactions, an extended electrostatic treatment was applied with the electrostatic interactions within 12 Å described by group-based coulombic interactions.

### Enzyme assays.

IMP, acetylpyridine adenine dinucleotide (APAD^+^), Tris, and MES were purchased from Sigma. DTT was purchased from Research Organics. Wild-type and Glu431Gln IMPDH from T. foetus were expressed in E. coli and purified as described previously [10,32]. All assays were performed at 25 °C. The release of NADH is partially rate limiting [11,31]. Therefore, to ensure that hydrolysis is completely rate limiting, these experiments used APAD^+^ [31]. Pre-steady-state experiments were performed to demonstrate that hydride transfer and APADH are rapid over the entire pH range ([11] and unpublished data). Standard IMPDH assays contained saturating concentrations of IMP (2 mM) and varying concentrations of APAD^+^ in 100 mM KCl, 1 mM DTT, and 50 mM of the appropriate buffer (MES for pH 5.0–7.0, and Tris-HCl for pH 7.3–9.3). Activity was measured by monitoring the absorbance of APADH at 363 nm on a Hitachi U-2000 UV-visible spectrophotometer. Steady-state parameters with respect to APAD^+^ were derived at saturating IMP concentrations by plotting the initial velocity against APAD^+^ concentration and fitting to an equation describing uncompetitive substrate inhibition using SigmaPlot (SPSS):

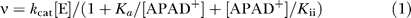


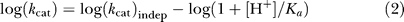
where (*k*_cat_)_app_ are the apparent values at each pH, (*k*_cat_)_indep_ are the pH-independent values, and *K_a_* is the acid dissociation constant for the most acidic ionization.

### Phylogenetic analysis.

IMPDH/GMPR amino acid sequences (IMPDH IPR005990, GMPR1 IPR005993, and GMPR2 IPR005994) were retrieved from the InterPro database (http://www.ebi.ac.uk/interpro/). Additionally, BLAST [44] searches with the T. foetus IMPDH (P50097) and human GMPR1 (P36959) amino acid sequences were performed. Sequences from the BLAST search that were already part of the InterPro dataset were removed, and an initial multiple sequence alignment was performed with MUSCLE [45]. A neighbor joining tree (unpublished data) was constructed in PAUP* 4.0b10 [46], and 95 sequences were selected for a Bayesian phylogenetic analysis. The sequences of this subset were realigned with Espresso [47,48]. A Bayesian phylogenetic analysis was performed with the parallel version of MrBayes 3.1.2 [49,50]. Amino acid substitution rates and state frequencies were fixed to the WAG parameters [51]. A uniform (0.0, 200.0) prior was assumed for the shape parameter of the gamma distribution of substitution rates [52], an unconstrained exponential prior with rate 10.0 for branch lengths, and all labeled topologies were a priori equally probable. Two independent MCMC analyses were run, each with one cold chain and three heated chains, with the incremental heating schema implemented in MrBayes (λ=0.2). Convergence was assumed after the topology samples from the two cold chains had reached an average standard deviation of split frequencies of less than 0.01 (after 1,610,000 generations). Accession numbers, detailed results, and the full tree are found in Text S1.

### Complementation assay for GMPR activity.

E. coli strain H1173 (*F-*, *guaC23*, *tonA2*, *proA35*, *lacY1*, *tsx-70*, *supE44?*, *gal-6*, *l-*, *trp-45*, *tyrA2*, *rpsL125*, *malA1 (lR)*, *xyl-7*, *mtl-2*, *thi-1*, *purH57*) contains mutations in *purH* and *guaC*, and therefore requires both adenosine and guanosine for growth. Bacteria were transformed with pGS682 carrying either the wild-type *guaC* gene or variants containing C186A, T188A, and E289Q mutations. Cultures were grown overnight in LB or LB/ampicillin and 5 μl of 1/20 serial dilutions were plated on M9 minimal media containing 0.5% casamino acids, 100 μg/ml l-tryptophan, 0.1% thiamin, 50 μg/ml guanosine, and/or 50 μg/ml adenosine.

## Supporting Information

Figure S1 Optimization of the Wang-Landau Metadynamics Conditions In order to optimize the Wang-Landau metadynamics conditions, three setups with the final Gaussian heights 1.0 kcal/mol, 0.06 kcal/mol, and 0.01 kcal/mol were executed. The 1.0 kcal/mol simulation yielded a result with large uncertainties and gave the free energy barrier of 14 kcal/mol at the end of a 1-ns simulation. The 0.01 kcal/mol simulation yielded a nicely converged free energy diagram with a barrier of 8 kcal/mol, but required more than 20 ns. The 0.06 kcal/mol also yielded a free energy barrier of 8 kcal/mol with acceptable fluctuations, but required only 5 ns. Based on these benchmark results, 0.06 kcal/mol was utilized as the final Gaussian height throughout all the simulations.
(3.5 MB TIF)

Figure S2 Simulations of the Thr Pathway with Glu431 Treated MM. Methods as described in Figure S1
(1.75 MB TIF)

Figure S3Experimental Estimation of the Contribution of the Thr PathwayAssuming that E431Q mutation disables the Thr pathway, but has no effect on the pH dependence of the Arg pathway, then the pH-rate profile of the wild-type enzyme is described by:

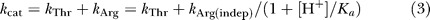
where *K_a_* = 10^−7.6^ as determined from the pH dependence of E431Q. The pH-rate profile of the wild-type enzyme could be reasonably described with the above equation when the value of *k*_Thr_ is 0.15 s^−1^, which corresponds to an energy barrier of approximately 19 kcal/mol. For easier visualization, the pH-rate profiles of Figure 3A were normalized so that the pH-independent values of *k*_cat_ = 1 for both wild type and E431Q, where *x* becomes the normalized value of *k*_Thr_.
(2.05 MB TIF)

Figure S4 Phylogenetic Tree of IMPDH and GMPR The unrooted tree was inferred with MrBayes (including posterior probabilities) [49]. Organism names are followed by their sequence accession codes. IMPDH, GMPR1, and GMPR2 refer to members of InterPro accession codes IPR005990, IPR005993, and IPR005994, respectively.
(4.39 MB TIF)

Text S1 Phylogenetic Analysis Detailed description of the derivation of Figure S4.
(101 KB PDF)

## Abbreviations

GMPR: GMP reductase
IMPDH: IMP dehydrogenase, MZP, mizoribine monophosphate
SIE: solvent isotope effect
