# Supplementary figures and images for: An Enzymatic Atavist Revealed in Dual Pathways for Water Activation
Source: PLoS Biol. 2008 Aug 26;6(8):e206. doi: 10.1371/journal.pbio.0060206 (PMC2525682; doi:10.1371/journal.pbio.0060206)

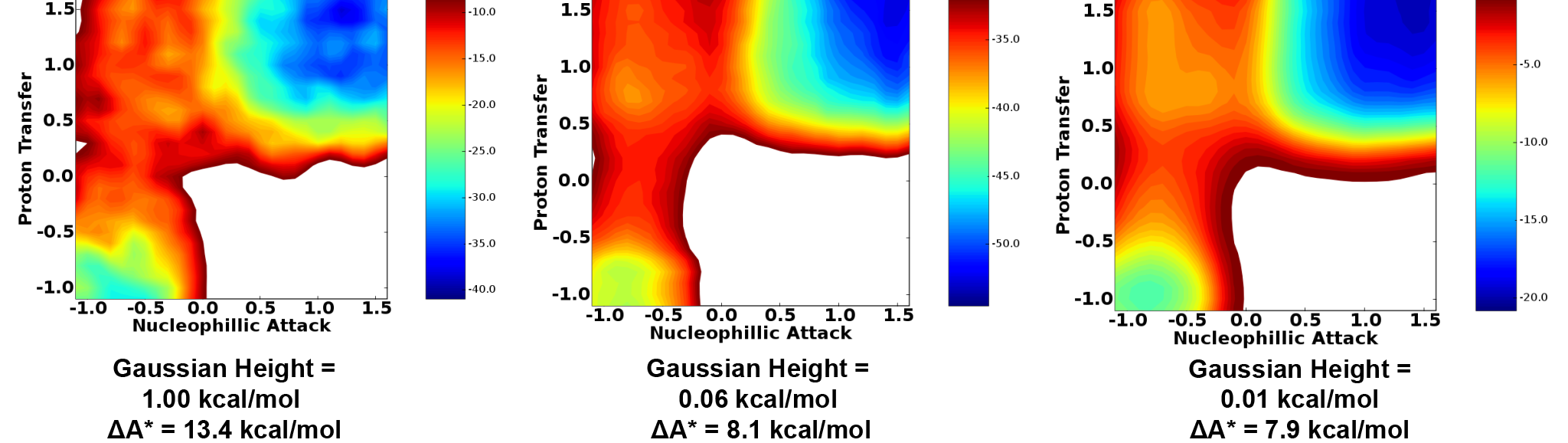

Supplement: Figure S1 — In order to optimize the Wang-Landau metadynamics conditions, three setups with the final Gaussian heights 1.0 kcal/mol, 0.06 kcal/mol, and 0.01 kcal/mol were executed. The 1.0 kcal/mol simulation yielded a result with large uncertainties and gave the free energy barrier of 14 kcal/mol at the end of a 1-ns simulation. The 0.01 kcal/mol simulation yielded a nicely converged free energy diagram with a barrier of 8 kcal/mol, but required more than 20 ns. The 0.06 kcal/mol also yielded a free energy barrier of 8 kcal/mol with acceptable fluctuations, but required only 5 ns. Based on these benchmark results, 0.06 kcal/mol was utilized as the final Gaussian height throughout all the simulations. (3.5 MB TIF) [file pbio.0060206.sg001.tif]

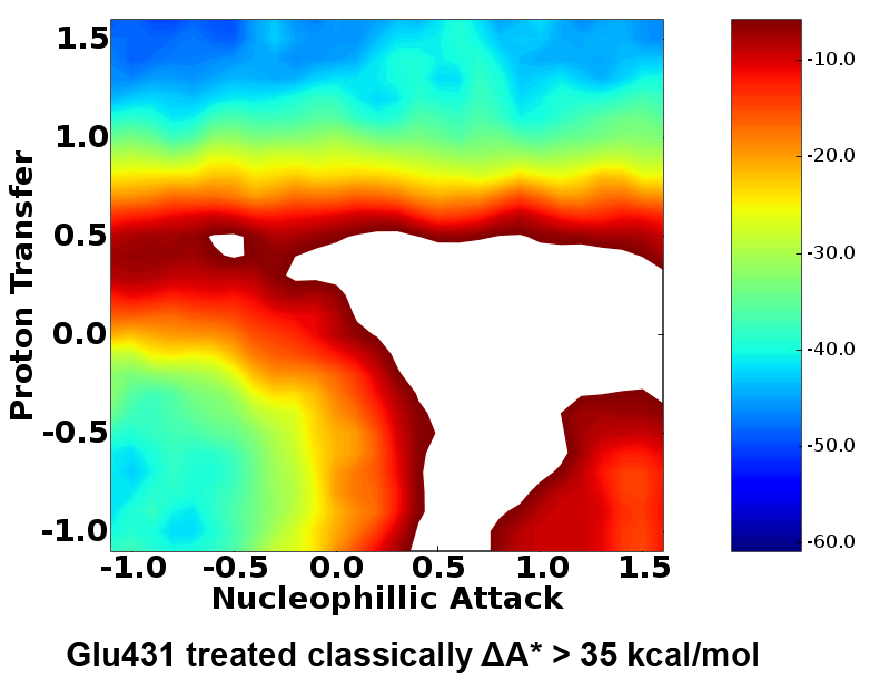

Supplement: Figure S2 — Methods as described in Figure S1 (1.75 MB TIF) [file pbio.0060206.sg002.tif]

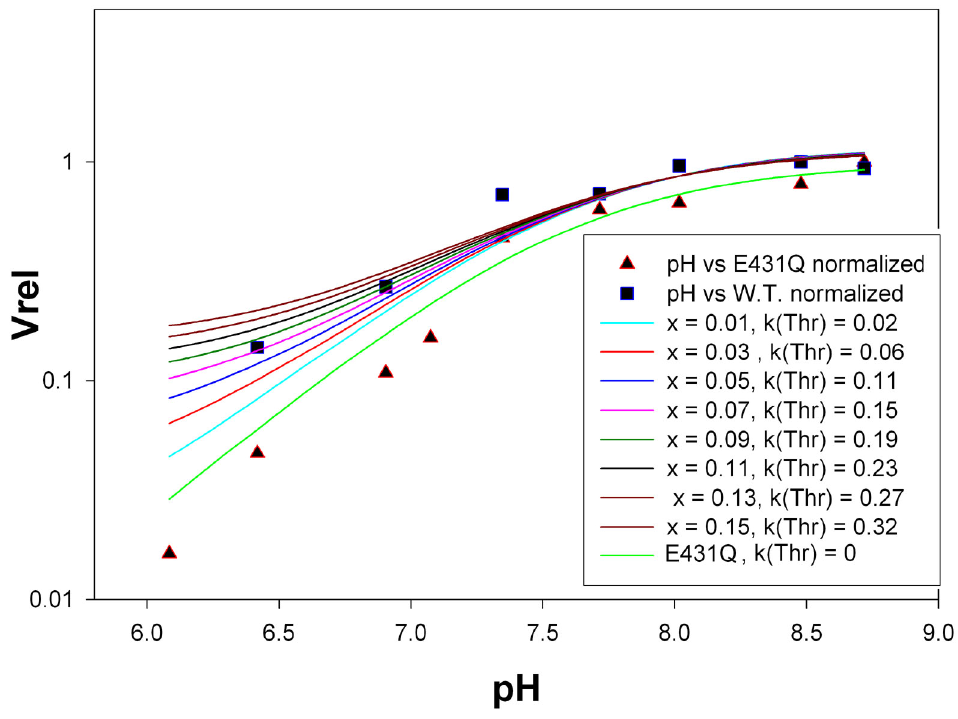

Supplement: Figure S3 — Assuming that E431Q mutation disables the Thr pathway, but has no effect on the pH dependence of the Arg pathway, then the pH-rate profile of the wild-type enzyme is described by: where Ka = 10−7.6 as determined from the pH dependence of E431Q. The pH-rate profile of the wild-type enzyme could be reasonably described with the above equation when the value of k Thr is 0.15 s−1, which corresponds to an energy barrier of approximately 19 kcal/mol. For easier visualization, the pH-rate profiles of Figure 3A were normalized so that the pH-independent values of k cat = 1 for both wild type and E431Q, where x becomes the normalized value of k Thr. (2.05 MB TIF) [file pbio.0060206.sg003.tif]

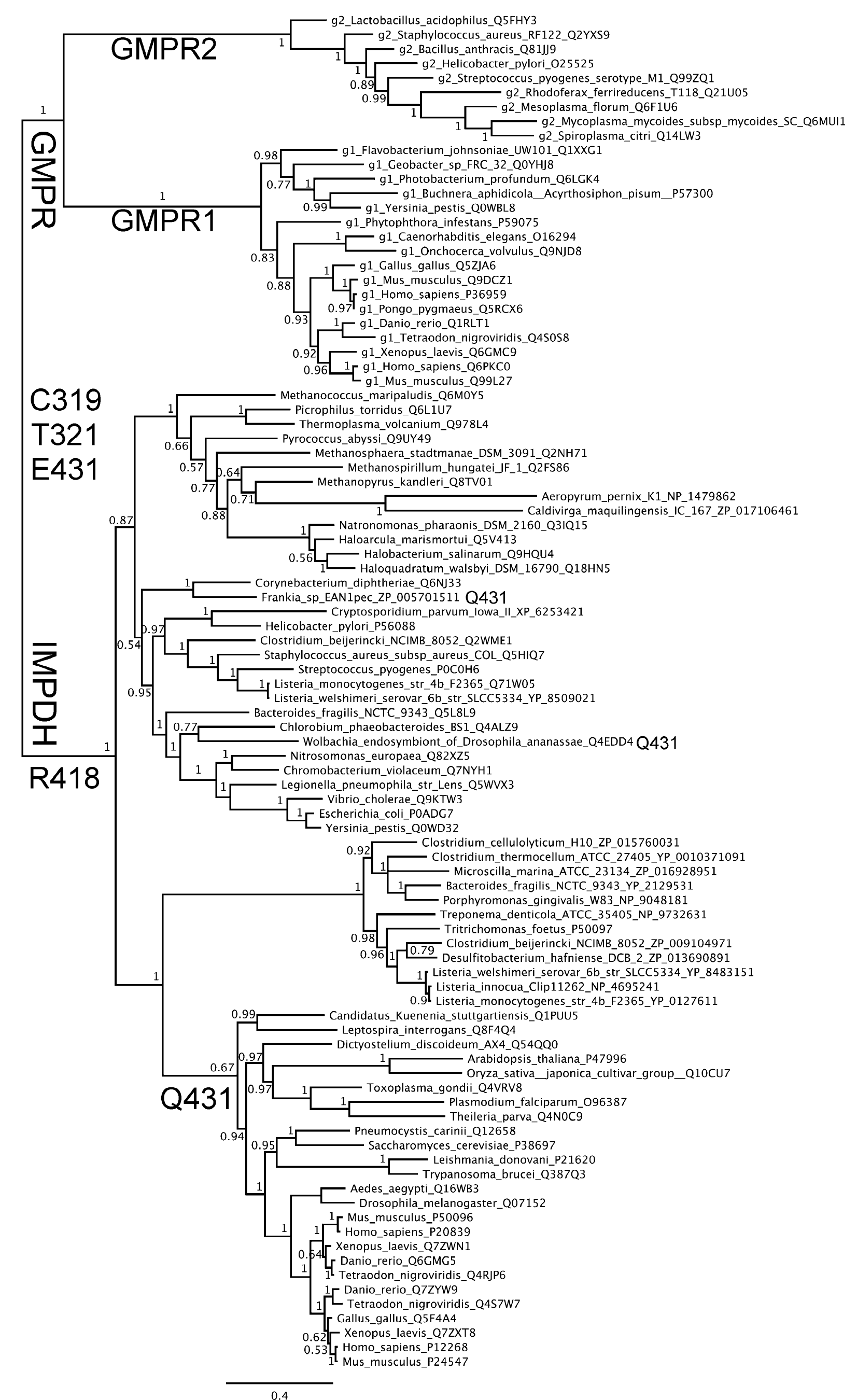

Supplement: Figure S4 — The unrooted tree was inferred with MrBayes (including posterior probabilities) [49]. Organism names are followed by their sequence accession codes. IMPDH, GMPR1, and GMPR2 refer to members of InterPro accession codes IPR005990, IPR005993, and IPR005994, respectively. (4.39 MB TIF) [file pbio.0060206.sg004.tif]
