# Supplementary material for: An Enzymatic Atavist Revealed in Dual Pathways for Water Activation
Source: PLoS Biol. 2008 Aug 26;6(8):e206. doi: 10.1371/journal.pbio.0060206 (PMC2525682; doi:10.1371/journal.pbio.0060206)
Supplement: Text S1 — Detailed description of the derivation of Figure S4. (101 KB PDF) [file pbio.0060206.sd001.pdf]

Text S1

Enzymatic Atavist Revealed in Dual Pathways for Water Activation

Donghong Min[1], Helen R. Josephine[2], Hongzhi Li[1], Clemens Lakner[1,3], Iain S. MacPherson[2], David Swofford[1,3], Lizbeth Hedstrom[2,4]* and Wei Yang[1,5,6]*

1. School of Computational Science, Florida State University, Tallahassee, FL, 32306
2. Department of Biochemistry, Brandeis University, Waltham, MA, 02454
3. Department of Biological Sciences, Florida State University, Tallahassee, FL, 32306
4. Department of Chemistry, Brandeis University, Waltham, MA 02454
5. Department of Chemistry and Biochemistry, Florida State University, Tallahassee, FL, 32306
6. Institute of Molecular Biophysics, Florida State University, Tallahassee, FL, 32306

*Correspondence: yang@sb.fsu.edu (W.Y.); hedstrom@brandeis.edu (L.H.)

Phylogenetic Analysis.

The unrooted tree, including posterior probabilities, was inferred with MrBayes (Figure S4) [1] using the following Interpro accession numbers: Parent, IPR001093 IMPDH/GMPR; Children, IPR005990 IMPDH, IPR005993 GMPR1, IPR005994 GMPR2. Note that the nomenclature is confusing because GMPR1 includes both eukaryotic GMPR type 1 and 2. The following child entries were not included in the analysis due to unknown function: IPR005991 IMPDH-related 1 and IPR005992 IMPDH-related 2. The sequence Q9GZH3 (*Caenorhabditis elegans* IMPDH) was omitted from the analysis because it's position at the base of the eukaryote branch is unstable. Probably as a result of gene duplication, two IMPDH genes were found in four bacterial species: *Bacteroides fragilis* NCTC 9343 (Q5L8L9, YP_212953), *Clostridium beijerincki* NCIMB 8052 (Q2WME1, ZP_00910497), *Listeria monocytogenes* str. 4b F2365 (Q71W05, YP_012761), and *Listeria welshimeri* serovar 6b (YP_848315, YP_850902).

IMPDH: Residues Cys319, Thr321, Arg418 and Tyr419 are conserved in all 444 sequences of IPR005990 as well as in any additional sequences from the BLAST search that grouped within this clade on a neighbor joining tree. Therefore the Arg pathway is present in all IMPDHs, as is the ability to form E-XMP*. In contrast, either Glu or Gln are observed at position 431. The tree is monophyletic for eukaryotic IMPDH, and all eukaryotic sequences (except *Tritrichomonas foetus* and the sequences from *Cryptosporidium parvum* and *C. hominis*) have Gln at position 431. Since the *T. foetus* and *Cryptosporidium* sequences contain Glu431 and are nested within bacterial sequences on the phylogenetic tree, it has been presumed that these genes were acquired by lateral gene transfer [2-4]. Virtually all bacteria contain Glu431. The exceptions include *Candidatus kuenenia stuttgartiensis* (Q1PUU5) and *Leptospira* (Q8F4Q4, YP_798134), which group in a separate clade neighboring eukaryotes. The adjacent clade contains Glu431 collectively, so according to this inference, the change occurred at the branch leading to the ancestor of (Eukaryotes (*Leptospira*, *Candidatus*)). *Wolbachia sp.* (Q4EDD4, Q4E883, Q5GSA9 and Q73IR4) and *Frankia sp.* (ZP_00570151) also contain Gln431, which suggests that this mutation has evolved independently on several occasions. In summary, the Arg pathway is present in all IMPDHs, but the Thr pathway has been lost in the branch leading to Eukaryotes/*Leptospira*, *Candidatus* as well as in other isolated bacterial IMPDHs.

We reconstructed the ancestral sequence of residue 431 for the nodes in question using the FASTML program under the WAG model with gamma distributed rates [5].

N14 Eukaryotes

p(Q)=0.999829 p(E)=0.000144716

N16 (*Leptospira, Candidatus*)

p(Q)=0.99847 p(E)=0.000960704 p(K)=0.000277648 p(H)=0.000115309 p(R)=0.000100206

N15 (Eukaryotes, (*Leptospira, Candidatus*))

p(Q)=0.998024 p(E)=0.0017308 p(K)=0.000142351

N17 Ancestor of (Eukaryotes, (*Leptospira, Candidatus*))

p(E)=0.846335 p(Q)=0.1304 p(D)=0.00955844 p(K)=0.00719895 p(A)=0.00145566 p(N)=0.00119693 p(R)=0.000945701 p(H)=0.000726329 p(S)=0.000708511 p(T)=0.000536639 p(P)=0.000325941 p(G)=0.000275554 p(V)=0.000127451

The other two nodes connected to N17:

N74 Ancestor of the clade that includes *T. foetus*

p(E)=0.999335 p(D)=0.000371073 p(Q)=0.000216552

N18

p(E)=0.97282 p(Q)=0.0179498 p(D)=0.00536355 p(K)=0.0021137 p(A)=0.00047754 p(N)=0.000325481 p(R)=0.00019397 p(S)=0.000192772 p(T)=0.000157851 p(H)=0.00014696

GMPR: Cys319 and Thr321 are conserved in all sequences of both GMPR1 and GMPR2. With one exception, Glu431 is also found in all members of both GMPR1 and GMPR2. The exception, *Staphylococcus aureus subsp. aureus* JH9 (Q1XYF3), contains Gln431, but this enzyme has not been characterized, so it is possible that this substitution is a sequencing artifact. An intriguing alternative explanation also exists: many *Staphylococcus* produce Gln-tRNA(Gln) by amidating mischarged Glu-tRNA(Gln), so it is possible that Glu is incorporated during translation, at least to the extent required to produce sufficient functional enzyme to support bacterial growth. Therefore the Thr pathway is intact in all GMPRs. As noted in the text, there is no need to activate water in the GMPR reaction, so the Thr pathway must have an alternative, possibly related function, such as protonating the ammonium leaving group. Members of GMPR1 contain a Tyr-Arg dyad in the flap, which is tempting to equate with the Arg48-Tyrr419 dyad of IMPDH. However, the flap region contains variable sequence lengths and are therefore difficult to align with confidence. More importantly, the Tyr-Arg dyad of GMPR1 interacts with the 2'-phosphate of NADPH, and therefore serves a function that is unique to GMPR, much like activation of water is unique to IMPDH. The flap region of GMPR2 are very heterogeneous and do not contain the Tyr-Arg dyad. These observations suggest that while the Thr pathway is a common feature of both IMPDH and GMPR, the Arg pathway is unique to IMPDH.

Accession codes of the data set used for the phylogenetic analysis

IMPDH: ZP_005701511, YP_8509021, ZP_017106461, NP_1479862, YP_2129531, ZP_009104971, ZP_015760031, YP_0010371091, ZP_013690891, NP_4695241, YP_0127611, YP_8483151, ZP_016928951, NP_9048181, NP_9732631;

IMPDH IPR005990: Q16WB3, Q07152, Q6GMG5, P50096, Q4RJP6, P20839, P12268, Q7ZWN1, Q7ZYW9, Q5F4A4, Q4S7W7, Q7ZXT8, P24547, Q1PUU5, Q12658, P38697, Q54QQ0, Q4VRV8, P47996, Q10CU7, P50097, Q9GZH3, Q5V413, Q3IQ15, Q9HQU4, Q18HN5, P21620, Q387Q3, Q8F4Q4, Q6M0Y5, Q8TV01, Q2NH71, Q9UY49, Q2FS86, Q6L1U7, Q978L4, O96387, Q4N0C9, Q5L8L9, Q71W05, Q6NJ33, Q2WME1, Q5HIQ7, P0C0H6, Q4ALZ9, Q5WVX3, P0ADG7, Q0WD32, Q9KTW3, Q82XZ5, Q7NYH1, Q4EDD4, 6253421, P56088;

GMPR1 IPR005993 P57300, Q0YHJ8, O16294, Q1RLT1, Q4S0S8, Q6GMC9, Q1XXG1, Q5ZJA6, P36959, Q9DCZ1, Q5RCX6, Q6PKC0, Q99L27, Q6LGK4, P59075, Q0WBL8, Q9NJD8;

GMPR2 IPR005994: Q81JJ9, Q5FHY3, Q2YXS9, Q99ZQ1, O25525, Q6F1U6, Q6MUI1, Q14LW3, Q21U05;

References.

1. Ronquist F, Huelsenbeck JP (2003) MrBayes 3: Bayesian phylogenetic inference under mixed models. Bioinformatics 19: 1572-1574.
2. Bapteste E, Philippe H (2002) The potential value of indels as phylogenetic markers: position of trichomonads as a case study. Mol Biol Evol 19: 972-977.
3. Striepen B, White MW, Li C, Guerini MN, Malik SB, et al. (2002) Genetic complementation in apicomplexan parasites. Proc Natl Acad Sci U S A 99: 6304-6309.
4. Huang J, Mullapudi N, Lancto CA, Scott M, Abrahamsen MS, et al. (2004) Phylogenomic evidence supports past endosymbiosis, intracellular and horizontal gene transfer in Cryptosporidium parvum. Genome Biol 5: R88.
5. Pupko T, Pe'er I, Hasegawa M, Graur D, Friedman N (2002) A branch-and-bound algorithm for the inference of ancestral amino-acid sequences when the replacement rate varies among sites: Application to the evolution of five gene families. Bioinformatics 18: 1116-1123.
